# Asymmetric vestibular stimulation reveals persistent disruption of motion perception in unilateral vestibular lesions

**DOI:** 10.1152/jn.00674.2016

**Published:** 2017-08-16

**Authors:** R. Panichi, M. Faralli, R. Bruni, A. Kiriakarely, C. Occhigrossi, A. Ferraresi, A. M. Bronstein, V. E. Pettorossi

**Affiliations:** ^1^Dipartimento di Medicina Sperimentale, Sezione di Fisiologia Umana, Università di Perugia, Perugia, Italy; ^2^Dipartimento di Specialità Medico-Chirurgiche e Sanità Pubblica, Sezione di Otorinolaringoiatria, Università di Perugia, Perugia, Italy; ^3^Academic Neuro-Otology, Centre for Neuroscience, Charing Cross Hospital, Imperial College London, London, United Kingdom

**Keywords:** motion perception, asymmetric rotation, unilateral vestibular lesion, dizziness, vestibular neuritis

## Abstract

Self-motion perception was studied in patients with unilateral vestibular lesions (UVL) due to acute vestibular neuritis at 1 wk and 4, 8, and 12 mo after the acute episode. We assessed vestibularly mediated self-motion perception by measuring the error in reproducing the position of a remembered visual target at the end of four cycles of asymmetric whole-body rotation. The oscillatory stimulus consists of a slow (0.09 Hz) and a fast (0.38 Hz) half cycle. A large error was present in UVL patients when the slow half cycle was delivered toward the lesion side, but minimal toward the healthy side. This asymmetry diminished over time, but it remained abnormally large at 12 mo. In contrast, vestibulo-ocular reflex responses showed a large direction-dependent error only initially, then they normalized. Normalization also occurred for conventional reflex vestibular measures (caloric tests, subjective visual vertical, and head shaking nystagmus) and for perceptual function during symmetric rotation. Vestibular-related handicap, measured with the Dizziness Handicap Inventory (DHI) at 12 mo correlated with self-motion perception asymmetry but not with abnormalities in vestibulo-ocular function. We conclude that *1*) a persistent self-motion perceptual bias is revealed by asymmetric rotation in UVLs despite vestibulo-ocular function becoming symmetric over time, *2*) this dissociation is caused by differential perceptual-reflex adaptation to high- and low-frequency rotations when these are combined as with our asymmetric stimulus, *3*) the findings imply differential central compensation for vestibuloperceptual and vestibulo-ocular reflex functions, and *4*) self-motion perception disruption may mediate long-term vestibular-related handicap in UVL patients.

**NEW & NOTEWORTHY** A novel vestibular stimulus, combining asymmetric slow and fast sinusoidal half cycles, revealed persistent vestibuloperceptual dysfunction in unilateral vestibular lesion (UVL) patients. The compensation of motion perception after UVL was slower than that of vestibulo-ocular reflex. Perceptual but not vestibulo-ocular reflex deficits correlated with dizziness-related handicap.

acute unilateral vestibular lesions (UVL), in humans typically due to vestibular neuritis, disrupt vestibular reflexes (including loss of gaze and postural stability), as well as self-motion perception, represented initially by an intense rotational sensation (vertigo) (Baloh 2003; Halmagyi et al. 2010). After 48–72 h symptoms subside and vestibular reflexes gradually recover through a process of “central vestibular compensation,” which includes plastic changes in neuronal excitability, up- and downregulation of neurotransmitter receptors, synaptic plasticity, and neuronal growth (Curthoys 2000; Dutia 2010; Smith and Curthoys 1989). In conjunction with the improvement of vestibular symptoms and reflexes, quality of life also improves (Bergenius and Perols 1999; Kim et al. 2008; Patel et al. 2016).

Despite these general trends, however, the degree of clinical recovery after a UVL is variable and unpredictable in individual patients. This variability depends less on the severity of the lesion, as measured with tests of vestibulo-ocular reflex (VOR) function [degree of canal paresis (Shupak et al. 2008) or head-impulse test abnormality (Palla et al. 2008, Patel et al. 2016)], and more on individual differences in neurological (Adamec et al. 2014), psychological (Godemann et al. 2004), and visual psychophysical factors (Cousins et al. 2014, 2017). However, the dependence of recovery on vestibularly mediated motion perception has been neglected, despite animal physiology (Angelaki and Cullen 2008) and human imaging studies (Dieterich 2007; Helmchen et al. 2009) showing significant contributions by widespread cortico-subcortical structures to central vestibular processing and lesion-induced neural plasticity.

Acute UVL are known to induce dissociation between vestibuloperceptual and VOR function (Cousins et al. 2013) but, as central compensation develops, these differences are reduced (Cousins et al. 2017). Recent experiments, however, suggest that perceptual-reflex dissociations can be induced in normal subjects exposed to asymmetric rotation (Panichi et al. 2011; Pettorossi et al. 2013, 2015; Pettorossi and Schieppati 2014). In the course of repetitive asymmetric rotations, VOR responses keep or improve symmetry. Notably, however, central adaptive mechanisms make self-motion perception progressively more asymmetric. We postulate that activation of such central mechanisms involved in asymmetric vestibular adaptation are likely to play a critical part during the adaptive process of compensation after unilateral labyrinthine lesions. Thus biases in the motion perception system may persist longer than VOR biases during the compensatory period and this perceptual error could be responsible for long-term symptom development. This is the general hypothesis examined here.

Therefore, we investigated self-motion perception acutely and during compensation after UVL by using an asymmetric rotation technique known to induce a bias in central vestibular circuitry of normal subjects (Panichi et al. 2011). We compared this with the responses to symmetric rotation because symmetric sinusoidal rotation (perhaps due to additional motion-predictive components) is unlikely to reveal long-term biases in vestibular motion perception. We also compared vestibuloperceptual responses (ultimately cortically mediated) to the VOR elicited with the same symmetric and asymmetric stimuli, and with conventional clinical caloric, head shaking, and subjective visual vertical (SVV) testing. Finally, to examine the hypothesis that abnormalities in vestibularly mediated motion perception may contribute to long-term dizziness, we assessed the patients’ subjective clinical recovery using the Dizziness Handicap Inventory (DHI; Jacobson and Newman 1990; Nola et al. 2010).

## MATERIALS AND METHODS

### Participants

Thirty male patients aged 24–55 yr (mean 42.80, SD 8.35) were enrolled in a prospective study in UVL: 24 right-handed, 6 left-handed, 12 with vestibular deficit in the left side and 18 in the right side. Thirty normal subjects (controls) were also examined, age 20–55 yr old, mean 39 ± 11.3; 25 right-handed, 5 left-handed. Since in our previous study on perceptual responses to asymmetric rotation in normal subjects (Pettorossi et al. 2013) male participants showed less variability than female, we examined only male subjects in the present study. Patients were first seen for acute dizziness between 1 January 2009 and 31 December 2013. Inclusion criteria were based on signs and symptoms of vestibular neuritis described by Strupp and Brandt (2009). No patient had additional auditory or central nervous system symptoms or signs. They all received methylprednisolone for 15 days and were encouraged to become progressively physically active as symptoms subsided. All patients performed the standard vestibular rehabilitation program offered in our hospital.

The patients were examined four times: approximately at 1 wk (acute phase) and at 4, 8, and 12 mo after the acute episode. The 30 control subjects were also tested four times at the same time intervals. In accordance with the Declaration of Helsinki, subjects provided written, informed consent and the protocol was approved by the ethics committee of the University of Perugia. Testing was performed by a different team to the one participating in data analysis, largely unaware of the research questions.

### Test of Self-Motion Perception: Stimulation Apparatus and Recording

#### Stimulation apparatus.

**Fig. 1.**
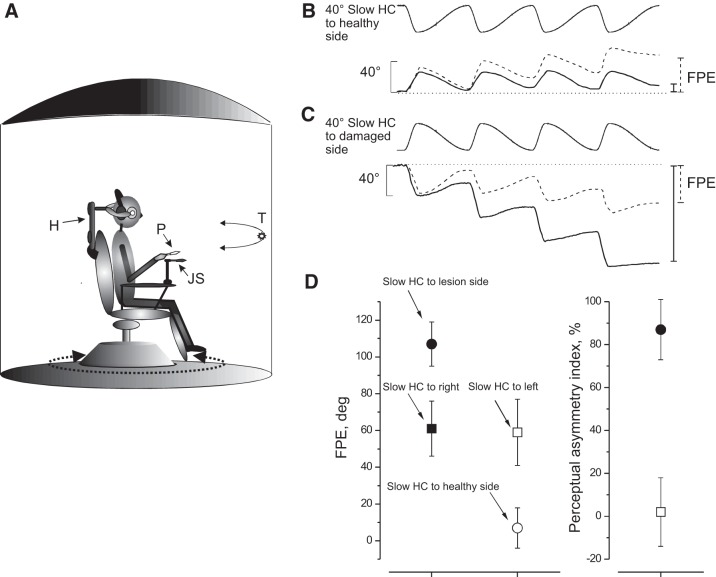
Experimental setting and motion perception recording. *A*: schematic drawing of the experimental setting. Acoustically isolated cabin and rotating chair: P, pointer; JS, joystick (when used); T, visual target, presented just before the rotation in the dark; H, head holder. *B* and *C*: motion perception tracking in response to opposite directed series of 4 asymmetric cycles with slow half cycle (Slow HC) to healthy side (*B*) and Slow HC to lesion side (*C*). Traces above show asymmetric rotation and those below show motion tracking during rotation [solid line, unilateral vestibular lesion (UVL) patient; dashed line, controls]. Final position error (FPE) at the end of rotation is indicated by vertical bars on the right (solid line, UVL patient; dashed line, controls). Note that FPE of UVL patients was smaller in *B* and greater in *C* than that of controls. *D*, *left graph*: FPE (mean and SD) of 30 normal (square) and 30 UVL patients (1 wk after acute episode) (circle) in response to opposite directed 2 series of 4 cycle of asymmetric rotation. *Left*: Slow HC to right (controls) and lesion side (UVL patients). *Right*: Slow HC to left or healthy side. Note a remarkable FPE difference in response to opposite directed asymmetric rotation. *Right graph*: perceptual asymmetry index (mean and SD) of UVL patients (●) and controls (□).

Subjects were seated on a rotating chair, within an acoustically isolated cabin, that rotated in the horizontal plane driven by a DC motor (Powertron, Contraves, Charlotte, NC) servo-controlled by an angular-velocity encoder (0.01–1 Hz, 1% accuracy). A head holder maintained the head 30° down tilted to align the horizontal semicircular canals with the rotational plane of the platform (Fig. 1). The trunk was tightly fastened to the chair. Roll and pitch head displacements were prevented by a plastic collar.

The chair oscillated sinusoidally at an amplitude of 40°. The vestibular stimulus used in the main experiment consisted of four cycles of asymmetric whole-body oscillations in the dark with the same back and forth amplitude, but different velocity (Pettorossi et al. 2013). The stimulus profile resulted from the combination of two sinusoidal half cycles of the same amplitude (40°) but different frequencies: fast half cycle (Fast HC) = 0.38 Hz and slow half cycle (Slow HC) = 0.09 Hz (Fig. 1). Peak acceleration during the Fast HC was 120°/s^2^ with peak velocity 47°/s, followed by the slow rotation in the opposite direction at a peak acceleration of 7°/s^2^, peak velocity 11°/s that returned the subject to the starting position. Both peak acceleration and velocity values are above thresholds for vestibular activation (Grabherr et al. 2008, Seemungal et al. 2004, Valko et al. 2012). As a control condition, before asymmetric stimulation, all subjects underwent symmetric rotation (40°, 0.38 and 0.09 Hz) to compare motion perception responses to the two stimuli.

#### Self-motion perception recordings.

We used a psychophysical tracking procedure to assess self-motion perception (Pettorossi et al. 2004; Siegle et al. 2009). Before starting the rotation, subjects fixated a target placed in front of them and were asked to continue to imagine the same target throughout the rotation in the dark with eyes closed. The target was a light spot (diameter 1 cm) projected onto the wall of the dark cabin in front of the subject at 1.5 m from the eyes. The spot was switched off just before rotation onset and switched back on after rotation had ended. Subjects were instructed to continuously track the remembered spot in the dark as if it was earth fixed by counterrotating the forearm partially flexed and adducted to orient a hand pointer (Fig. 1) toward the remembered spot. The pointer had a laser beam that was turned on at the end of rotation for measuring the angular distance between the projection of the beam on the wall and the real target position (final position error, FPE). All subjects reported that the procedure felt simple and intuitive. It could be argued that the voluntary pointing task we adopted may not truly reflect vestibularly mediated self-motion perception. However, similar pointing tasks using a vestibular-remembered saccade task (Bloomberg et al. 1991; Nakamura and Bronstein 1995) break down completely in humans devoid of vestibular function. Indeed, the results presented here in patients with unilateral vestibular lesions also indicate that the task is vestibularly mediated.

During asymmetric rotation, subjects perceive the Fast HC more vividly than the Slow HC (Pettorossi et al. 2013) and so, at the end of each session, the target is erroneously represented in the direction of the Slow HC (Fig. 1). This FPE results from the algebraic sum of single cycle errors plus additional adaptation that further enhances the perception of the fast rotation and reduces that of the slow rotation (Pettorossi et al. 2013). To assess these mechanisms in UVL patients, we delivered two rounds of “four-cycle asymmetric stimulation”: one with the Slow HC toward the lesion side and another session with the Slow HC toward the healthy side. These oppositely directed asymmetrical rotations were randomly assigned in direction and administered with an interval of 1 day. Since asymmetric rotation can induce long-lasting aftereffects (Pettorossi et al. 2015; Pettorossi et al. 2013), we ascertained in preliminary experiments in three UVL patients that 1-day rest interval was sufficient to prevent any carry over effects. The FPEs of the two four-cycle rotations were compared and the asymmetry index of the two FPEs for perceptual responses was evaluated as follows: (FPE toward lesion − FPE away from lesion)×100/the sum of the two FPEs.

For separately evaluating the perception to the Slow and Fast HCs and comparing with data from previous studies (Pettorossi et al. 2013) we recorded both perceptual tracking and the VOR in 10 controls and 10 UVL patients chosen for their superior tracking ability, as shown by the similarity of their tracking to the shape of the stimulus, that is, without jerks and pauses. In these 10 patients and 10 control subjects, we continuously recorded online tracking by connecting the chair-fixed pointer to a precision potentiometer (joystick, Fig. 1*A*), for detailed analysis as in previous experiments (Pettorossi et al. 2013). Cumulative amplitude of the motion perception during Fast and Slow HCs were separately computed by adding four half cycle responses of two rounds of “asymmetric rotations,” with the Slow HC in opposite directions (i.e., toward or away from the lesion). In these subjects the VOR was also recorded with bitemporal DC-EOG (DC-electrooculogram) (Pettorossi et al. 2013) in a separate session 1 day apart. Quick nystagmic components were removed offline and the cumulative amplitude of the four half cycles slow-phase eye movement (cumulative slow-phase eye position, SPEP) (Bloomberg et al. 1991; Popov et al. 1999) were computed for fast and slow rotation. The asymmetry in the VOR (asymmetry index) was also evaluated after two oppositely directed rounds of four-cycle asymmetric rotations with the formula (lesion-Slow HC cumulative SPEP−healthy-Slow HC cumulative SPEP)×100/the sum of the two SPEPs. Signals from the pointer, EOG, and chair motion were digitized by a 12-bit analog-to-digital card (LabVIEW, National Instrument) at a sampling rate of 500 Hz and stored for offline analysis.

### Conventional (Clinical) Vestibular Tests

#### Caloric test.

Irrigation was performed with the patient supine and the head raised 30° according to Fitzgerald-Hallpike method, with water at 44°C and subsequently at 30°C for 40 s, 5 min apart. We measured peak slow-phase eye velocity 60–90 s after irrigation onset and applied the Jongkees formula for canal paresis [(right cold + right warm) – (left cold + left warm)]×100/(right warm + left cold + left warm + right cold)] and for directional preponderance [(right warm + left cold) – (right cold + left warm)×100/(right warm + left cold + left warm + right cold)] (Jacobson et al. 1995; Jongkees et al. 1965).

#### Head shaking nystagmus test.

With the patient seated and the head flexed 30°, the head was passively rotated horizontally by ±45° at 1 Hz for 20 s, followed by EOG recording of any evoked nystagmus. The head shaking test was considered positive if the head shaking induced at least 2 clear post rotation nystagmic beats with a peak slow-phase eye velocity > 5°/s (Kamei et al. 1964).

#### Subjective visual vertical.

We used a faintly illuminated LED bar which was placed in a darkened room and set 1.50 m away, straight ahead from the seated subjects. The bar (30 × 1 cm) was mounted on the wall via a central rod, around which the bar could be rotated by remote control in both directions. At the upper end, there was a pointer that slid with the bar on a graduated scale (Faralli et al. 2007), in which 0° corresponds to perfect alignment with gravity. The bar was presented tilted 45° to the right or left and subjects realigned the bar to perceived verticality. Three measurements per side were conducted alternating the starting position from 45° right and left tilt. The mean value of the six measurements will be reported. In accordance with the literature mean values of SVV between +2° and −2° were considered normal (Friedmann 1970).

#### DHI.

Clinical outcome at 12 mo was assessed with the DHI (Jacobson and Newman 1990, Nola et al. 2010). The DHI comprises 25 items (questions) and three possible replies: “yes” (4 points), “sometimes” (2 points), and “no” (0 points) designed to access a patient’s functional (nine questions), emotional (nine questions), and physical (seven questions) limitations. A score <10 points indicates no handicap, scores between 16 and 34 points indicate mild handicap, 36–52 moderate, and 54–100 points severe handicap.

### Protocol

First, subjects were studied for the SVV, the caloric test, and the head shaking test. Then subjects underwent a training session with the tracking system to be used in the self-motion perception experiment. To this end, subjects were trained to track with the forearm the remembered target during symmetric and asymmetric rotation. For quality control of the manual tracking, a video camera placed in the cabin monitored the displacement of an infrared marker placed on the distal forearm. We stopped the training when visual inspection of the tracking showed no further improvement and good matching of the stimulus waveform. Depending on the subject, 5–10 trials were sufficient. The day after the subjects underwent the test for self-motion perception. We discarded responses when arm movements during rotation showed discontinuity, jerks, or pauses; inadequate tracking occurred in few subjects (four) and in few trials (1 or 2 trials).

### Data Evaluation and Statistical Analysis

The responses were statistically analyzed by a generalized mixed model analysis (GLM) with FPE asymmetry values or cumulative and single half cycle amplitude of the responses as the dependent variables and group (patients and controls), direction of the asymmetric rotation, time of testing, and interactions as the fixed effects of main interest and a random effect for the repeated measures. This analysis allowed to establish the statistical significance of the difference observed in patients and controls at different times of observation to find out whether and when patient values recovered to control values. The data collected from subjects who dropped out have been included (three patients and two normal subjects at the third assessment and another patient and normal subject at the fourth assessment dropped out of the study). Statistical post hoc analysis was performed with Bonferroni’s post hoc test for multiple comparisons. The level of significance was set at *P* < 0.05 for both the GLM values and post hoc comparisons. Prior to GLM, the Shapiro-Wilk test assessed normality and the Levene’s test of homogeneity of the variances. Linear correlations between self-motion perception asymmetry and other clinical tests were established at different times from the acute UVL episode to compare perceptual and reflex recovery. In addition, we examined the correlation between DHI score and self-motion perception asymmetry or canal paresis asymmetry at 12 mo from the acute episode. Exponential functions were used for fitting correlations and time courses of the various parameters measured during the process of vestibular compensation. *R* and χ^2^ values are reported for the goodness of linear and exponential fit.

The confidence interval (CI) of the values of vestibular tests was evaluated as 95% of the values resulting from our control group. All statistical evaluations and fittings were performed with the software OriginPro (Origin Laboratory, Northampton, MA) and SPSS 16.0 IBM in Armonk, NY.

## RESULTS

The main finding is that asymmetric rotation reveals a profound and long-lasting effect in self-motion perception in the UVL group. A large FPE asymmetry developed, due to a reduced turning perception when the Slow HC was directed toward the lesion side. This perceptual long-lasting defect is selective as it was not observed during symmetric rotation, nor in the VOR during asymmetric rotation. We present, first, the results of the whole patient group (*n* = 30), followed by the detailed cycle-by-cycle assessment of 10 patients and controls for further mechanistic insight, ending with a comparison of these data with clinical vestibular results.

### Self-Motion Perception During Asymmetric and Symmetric Rotation in Normal Subjects

**Fig. 2.**
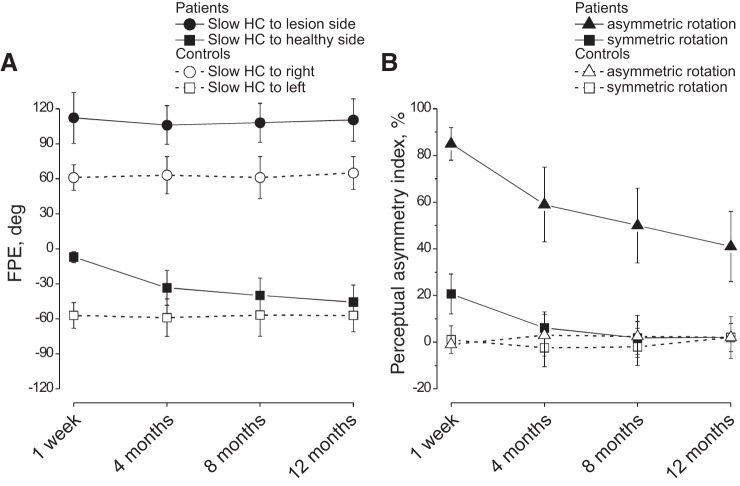
Final position error (FPE) and perceptual asymmetry index in UVL patients and controls in response to opposite directed asymmetric and symmetric rotations *A*: FPE (mean and SD) of the remembered target after 4 asymmetric rotation with Slow HC to lesion side (●) and to healthy side (■) from 30 patients at different time after the acute episode (1 wk; 4, 8, and 12 mo). Open symbols and dashed line indicate the FPE in response to opposite 4 cycle rotations in controls. The positive FPE is in the direction of Slow HC. Note that the amplitude of FPE in response to lesion-side Slow HC asymmetric rotation remains steady larger than in controls. Conversely, the FPE in response to healthy-side Slow HC rotation was acutely close to zero, but it increased over time toward the value of controls. *B*: mean and SD of the perceptual asymmetry index in UVL patients (solid symbols, solid line) and in controls (open symbols, dashed line) after asymmetric (triangle) and (0.09 Hz) symmetric (square) rotation. The perceptual asymmetry was very large and persisted for asymmetric rotation, while it was detectable only at the first measure for symmetric rotation.

As reported in previous papers (Panichi et al. 2011; Pettorossi et al. 2013), the tracking position of the remembered visual target at the end of four asymmetric rotation cycles (final position error, FPE) was shifted by circa 60° in the direction of the Slow HC (Fig. 1, *B* and *C*), with minimal right-left asymmetry (Fig. 2*B*) [perceptual asymmetry index = (right FPE−left FPE)×100/(right FPE + left FPE)]. This remained unchanged when reexamined at 4, 8, and 12 mo (Fig. 2B). During symmetric rotation, both FPE and degree of asymmetry were close to 0°.

### Self-Motion Perception During Asymmetric and Symmetric Rotation in UVL Patients

#### Motion perception during asymmetric rotation.

In UVL patients the magnitude of the FPE depended on whether the Slow or Fast HCs were directed toward the lesion or healthy side. The FPE induced by the Slow HC directed toward the lesion side (lesion-Slow HC) was significantly higher than that of controls at all recording stages (grouped analysis: *P* = 0.0007) (Fig. 1, *B* and *C*; Fig. 2*A*, Table 1). In contrast, FPE during rotation with the Slow HC toward the healthy side (healthy-Slow HC) was significantly lower than that in controls (grouped analysis: *P* = 0.003). Across time, the lesion-Slow HC FPE remained unchanged (*P* = 0.1–0.8), whereas the healthy-Slow HC FPE significantly increased, reaching control values at 12 mo (*P* = 0.31). The FPE asymmetry index of patients was very large at 1 wk (85.4 ± 7.8%) and remained significantly higher than in controls even at 12 mo (41.9 ± 15.4%) (Figs. 1*D* and 2*B* and Table 2).

**Table 1.** Statistical data for comparing FPE values in patients and controls, in response to opposite asymmetric side rotation at different testing time

FPE in patients and controls	df	*F*	*P*	Partial η^2^
group (patients vs. controls)	1,102	46.5	<0.001	0.33
side rotation	1,102	449.3	<0.001	0.81
time	3,306	18.12	<0.001	0.15
group×side rotation	1,102	470.2	<0.001	0.82
group×time	3,306	19.7	<0.001	0.16
side rotation×time	3,306	16.2	<0.001	0.14
group×side rotation×time	3,306	14.6	<0.001	0.13

Generalized mixed model analysis (GLM) of final position error (FPE) of 30 patients and 30 normal subjects after 4 cycles of opposite directed asymmetric rotations. Group, patients vs. controls; side rotation, lesion-slow half cycle (SHC) vs. healthy-SHC rotation; time, testing time. Size effect: partial η^2^.

**Table 2.** Statistical data for comparing the FPE asymmetric index in patients and controls in response to asymmetric and symmetric rotations at different testing time

	df	*F*	*P*	Partial η^2^
*Asymmetric index after asymmetric rotation in patients and controls*				
Groups	1,51	406.1	<0.0001	0.92
Time	3,153	69.3	<0.0001	0.57
Group×Time	3,153	67.4	<0.0001	0.56
*Asymmetric index after symmetric rotation (0.09 Hz) in patients and controls*				
Groups	1,51	16.5	=0.08	0.059
Time	3,153	6.6	=0.009	0.13
Group×Time	3,153	8.3	=0.003	0.21
*Asymmetric index after symmetric rotation (0.4 Hz) in patients and controls*				
Groups	1,51	15.9	=0.07	0.048
Time	3,153	5.8	=0.02	0.09
Group×Time	3,153	7.4	=0.004	0.18

GLM analysis of asymmetric index of 30 patients and 30 normal subjects after 4 cycles of opposite directed asymmetric and symmetric 0.09 and 0.4 Hz rotations. Group, patients vs. controls; time, testing time. Size effect: partial η^2^.

#### Motion perception during symmetric rotation.

The patients were also tested with symmetric sinusoidal rotation at 0.09 and 0.38 Hz (the frequencies of the asymmetric half cycles). FPE asymmetry between the healthy/lesion sides was minimal compared with the asymmetry present following asymmetric rotation, and not significantly different from control values in response to 0.09 Hz (*P* = 0.08) and to 0.38 Hz (*P* = 0.07) (statistical data: Table 2). *S*pecifically, the patient vs. control asymmetric index in response to symmetric rotation was significantly different only in the acute phase (for 0.09 and 0.38 Hz, *P* = 0.04–0.02) but not at 4 (*P* = 0.2 – 0.43), 8 (*P* = 0.3 – 0.5), or 12 (*P* = 0.23 – 0.3) mo (Fig. 2*B*).

### Online Recording During Symmetric and Asymmetric Rotation in 10 UVL Patients: Self-Motion Perception and VOR

#### Self-motion perception.

**Fig. 3.**
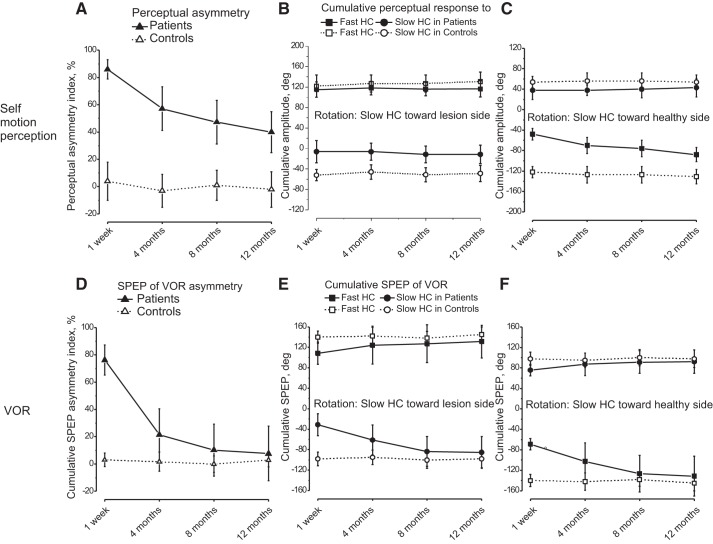
Cumulative amplitude of Fast HC response and Slow HC response to asymmetric rotation: different effect on self-motion perception (*A*–*C*) and vestibulo-ocular reflex (VOR; *D*–*F*). *A*: asymmetry index of self-motion perception in 10 patients (▲) after the acute unilateral vestibular lesion (UVL) and 10 control subjects (△). *B* and *C*: cumulative amplitude (mean and SD) of Fast HC response (squares) and Slow HC response (circles) after 4 cycle asymmetric rotation toward lesion-Slow HC (*B*) and healthy-Slow HC (*C*) at different time after UVL in patients (solid symbols and solid line) and control subjects (open symbols and dashed line). Note that the Slow HC response for lesion-Slow HC rotation is markedly reduced and a significant change during compensation period occurs only in response to the Fast HC directed toward the lesion side. *D*: asymmetry index of the cumulative slow-phase eye position (SPEP; ▲, patients; △, controls) after the acute UVL. Cumulative amplitude of SPEP of VOR asymmetries (*E* and *F*). Amplitude (mean and SD) of Fast HC responses (squares) and Slow HC responses (circles) after lesion-Slow HC (*E*) and healthy-Slow HC (*F*) asymmetric rotation at different intervals after UVL in patients (solid symbols and solid line) and controls (open symbols, dashed line) from 10 patients. Note that the asymmetry of VOR in response to asymmetric rotation markedly diminished during compensation period (*D*) due to the fact that both the amplitude of slow-phase eye response to Slow HC during lesion-Slow HC rotation (*E*) and that to FHC during healthy-Slow HC rotation (*F*) increased.

We analyzed the separate contribution of the Slow and Fast HCs responses to self-motion perception asymmetry in 10 patients and in 10 control subjects, selected on the basis of their better tracking ability (see materials and methods). These 10 UVL patients were rather homogenous regarding the degree of vestibular damage both at the acute and 12-mo stages, with values of canal paresis of 68 ± 8 and 10 ± 6% at 12 mo, respectively. The values at 12 mo were within the normal range (Fig. 6). Despite this good peripheral vestibular recovery, these patients nevertheless showed a persistent perceptual bias, not different from that observed in the patient group as a whole: groups (whole patient and patient subgroup) (*F*_1,34_ = 0.51, *P* = 0.62, partial η^2^ = 0.12), time (*F*_3,102_ = 54.1, *P* = 0.006, partial η^2^ = 0.63), time×groups (*F*_3,102_ = 0.34, *P* = 0.45, partial η^2^ = 0.13) (Figs. 2 and 3*A*).

The cumulative perceptual responses of the Slow HC and Fast HC (Fig. 3, *B* and *C*) were measured during the oppositely directed asymmetric rotations with Slow HC toward the lesion side (lesion-Slow HC) and Slow HC toward the healthy side (healthy-Slow HC). This analysis allowed to examine in a more homogeneous group the contribution of the slow and fast responses in the origin of the FPE asymmetry observed in the whole patient group. We found that the Slow HC responses directed toward the lesion side were responsible for the large persistent perceptual asymmetry since these responses remained very low up to 12 mo (statistical data are reported in Table 3). The cumulative Slow HC responses to rotation toward the lesion side were smaller than those of controls and they persisted over time (Slow HC responses vs. controls at all the recording time: *P* < 0.001 and the responses at 12 mo: *P* < 0.001). Conversely, the Slow HC responses toward the healthy side were slightly lower than those of controls (Slow HC responses vs. controls: *P* = 0.015), but at 12 mo were not significantly different from the controls (*P* = 0.074).

**Table 3.** Statistical data for comparing separate cumulative perceptual responses to slow and fast rotation in patients and controls after opposite directed asymmetric rotations at different testing time

	df	*F*	*P*	Partial η^2^
*Cumulative perceptual response to Slow HC rotation*				
groups	1,36	26.9	<0.001	0.14
direction	1,36	114.2	<0.001	0.33
time	3,108	3.5	=0.05	0.90
group×direction	1,36	114	<0.001	0.29
group×time	3,108	4.1	=0.005	0.10
direction×time	3,108	6.3	=0.003	0.39
group×direction×time	3,108	8.4	<0.001	0.41
*Cumulative perceptual response to Fast HC rotation*				
groups	1,36	21.1	<0.001	0.17
direction	1,36	130.2	<0.001	0.41
time	3,108	3.5	=0.004	0.87
group×direction	1,36	122	<0.001	0.32
group×time	3,108	4.1	=0.005	0.11
direction×time	3,108	7.3	=0.002	0.46
group×direction×time	3,108	5.3	<0.001	0.48

GLM analysis of 10 patients and 10 normal subjects of cumulative amplitude of the perceptual responses to slow half cycle (Slow) and fast half cycle (Fast HC). Group, patients vs. controls; direction, lesion vs. healthy side asymmetric rotation; time, testing time. Size effect: partial η^2^.

Also, the cumulative Fast HC responses toward the lesion side was smaller than those of control acutely (*P* = 0.0012), but thereafter increased up to 12 mo reaching values not different from the controls (at 12 mo: *P* = 0.064). Conversely, the cumulative amplitude of Fast HC response toward the healthy side was not significantly different compared with the control at any time (*P* = 0.29–0.49).

**Fig. 4.**
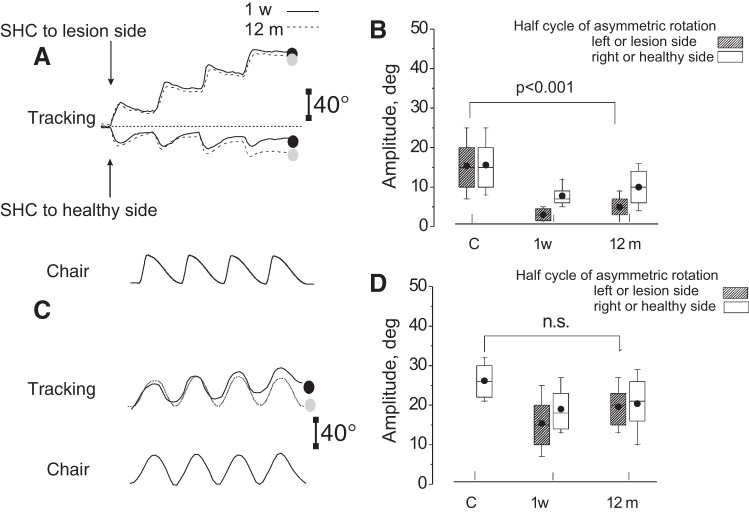
Continuous recording of motion perception in response to asymmetric and symmetric rotation in 10 patients in the early and late phase of the compensation period. *A* and *C*: tracking of motion perception in response to opposite directed series of 4 asymmetric cycles (A) with Slow HC (SHC) to lesion side (upper traces, ↑) or to healthy side (lower traces, ↓) and to symmetric rotation (0.09 Hz) (*C*). In *A* and *C*, solid line represents 1 wk after the acute UVL episode; and dashed line 12 mo after. Vertical bar: calibration of tracking. *Bottom* of *A* and *C* shows the chair rotation. Note the large difference in the final position error which depends on the direction of asymmetric rotation (FPE, black spot: at 1 wk, and FPE, gray spot: at 12 mo after the acute UVL episode). The difference persists in the asymmetric rotation over time, while in symmetric rotation there is no FPE in the late measure. *B* and *D*: graphs of the amplitude (in degrees) of the perceptual responses to the last Slow HC asymmetric (*B*) and symmetric rotation (0.09 Hz) (*D*). Box, whisker, horizontal line, and circle represent range, quartile, median, and mean, respectively. Dashed box, Slow HC rotation to left or lesion side; white box, Slow HC rotation to right or healthy side. The response obtained from the controls (C) and in UVL patients at 1 wk (1 w) and 12 mo (12 m) from the acute episode. Note the significant difference of the Slow HC of the asymmetric rotation between controls and UVL patients, when slow rotation is toward the lesion side. This difference is not present in the half cycle symmetric rotation at 12 mo. GLM statistical analysis: asymmetric Slow HC to lesion: groups (patients and controls) (*F*_1,18_ = 150.2, *P* < 0.001, partial η^2^ = 0.86), time (*F*_1,18_ = 0.84, *P* = 0.35, partial η^2^ = 0.11), groups×time (*F*_1,18_ = 0.65, *P* = 0.68, partial η^2^ = 0.11); asymmetric Slow HC to healthy side: group (*F*_1,18_ = 7.3, *P* < 0.02, partial η^2^ = 0.35), time (*F*_1,18_ = 2.6, *P* = 0.12, partial η^2^ = 0.25), groups×time (*F*_1,18_ = 1.2, *P* = 0.26, partial η^2^ = 21); symmetric Slow HC to lesion side: groups (patients and controls) (*F*_1,18_ = 5.2, *P* < 0.05, partial η^2^ = 0.50), time (*F*_1,18_ = 0.24, *P* = 0.38, partial η^2^ = 0.41), groups×time (*F*_1,18_ = 0.35, *P* = 0.46, partial η^2^ = 0.42); symmetric Slow HC to healthy side: groups (patients and controls) (*F*_1,18_ = 4.6, *P* < 0.05, partial η^2^ = 0.22), time (*F*_1,18_ = 0.45, *P* = 0.7, partial η^2^ = 0.24), groups×time (*F*_1,18_ = 0.58, *P* = 0.59, partial η^2^ = 0.11).

A cycle-by-cycle analysis of the responses to the Slow HC showed a gradual decrease in response amplitude both toward and away from the lesion side with the response to the last cycle being minimal and, in some patients, null (Fig. 4*A*). However, the reduction was greater when the slow rotation was toward the lesion side (decrease compared with the first cycle: 53 ± 15%) than toward the healthy side (28 ± 17%) (*F* = 37.41, *P* = 0.003).

Additional mechanistic insight is provided by comparing Slow HC perceptual responses to the asymmetric and symmetric (0.09 Hz) rotation (Fig. 4 and statistical data in the figure legend). During asymmetric rotation the perceptual responses to the slow rotation were depressed in the early and late measures. At 12 mo the responses were very low in comparison with the control (*P* < 0.001), mostly when directed toward the lesion side. Conversely, the amplitude of the perceptual response to symmetric rotation was reduced only acutely. At the late measure the gain in both directions recovered to normal (*P* = 0.3).

#### VOR.

In the same 10 patients and 10 control subjects the cumulative SPEP of the VOR was examined. The cumulative SPEP, obtained by removing the quick phases of the nystagmus and measuring the final offset in eye position, can be considered equivalent to the cumulative perceptual tracking response. One week after the acute event, the SPEP during four cycles of asymmetric rotation with the Slow HC toward the lesion side accumulated a large position error **(**76 ± 16°) whereas with the Slow HC toward the healthy side the error was negligible (5 ± 7°). At 12 mo, the final cumulative position error was 42 ± 9° for Slow HC toward the lesion side and 35 ± 13° for Slow HC toward the healthy side. As a consequence, the VOR SPEP asymmetry index decreased to 8.5 ± 19%. Therefore, the VOR asymmetric index was significantly different from the perceptual asymmetry index (Fig. 3, *A* and *D*) (perception and VOR, *F*_1,18_ = 34, *P* = 0.009, partial η^2^ = 0.12), time (*F*_3,54_ = 58, *P* = 0.0007, partial η^2^ = 0.12), time×perception, and VOR (*F*_3,54_ = 25, *P* = 0.0012, partial η^2^ = 0.12). Except for the first measure (*P* = 0.53), the perception asymmetry index was always significantly larger than that of the VOR response (*P* < 0.001). Notably, at 12 mo the perceptual asymmetry index was still ∼50% while the VOR asymmetry index was negligible (Fig. 3, *A* and *D*).

We analyzed separately the cumulative SPEP in response to Slow and Fast HC rotations (Fig. 3, *E* and *F*) (statistical comparison: Table 4). There were significant differences between patients and controls. In particular, the cumulative Slow HC and Fast HC SPEPs during rotation toward the lesion side and toward the healthy side were, as expected, smaller than those of controls (*P* < 0.001) at the first (acute) testing session, but they progressively increased reaching values not significantly different compared with controls at 8 mo (cumulative SPEP of patients vs. controls: *P* = 0.42–0.61) for lesion side rotation and at 4 mo (*P* = 0.14–0.32) for healthy side rotation.

**Table 4.** Statistical data for comparing separate cumulative SPEP of vestibulo-ocular reflex in response to slow and fast rotation in patients and controls after opposite directed asymmetric rotations at different testing time

	df	*F*	*P*	Partial η^2^
*Cumulative SPEP response to Slow HC rotation*				
groups	1,36	46.9	<0.001	0.19
direction	1,36	84.2	<0.001	0.38
time	3,108	13.5	=0.008	0.88
group×direction	1,36	105	<0.001	0.22
group×time	3,108	15.1	=0.004	0.15
direction×time	3,108	5.4	=0.006	0.42
group×direction×time	3,108	9.6	<0.001	0.49
*Cumulative SPEP response to Fast HC rotation*				
groups	1,36	12.9	<0.001	0.21
direction	1,36	142.2	<0.001	0.54
group×direction	1,36	101	<0.001	0.42
time	3,108	23.5	=0.001	0.77
group×time	3,108	7.1	=0.007	0.15
direction×time	3,108	9.3	=0.003	0.66
group×direction×time	3,108	19.43	<0.001	0.58

GLM analysis of 10 patients and 10 normal subjects of cumulative amplitude of the slow-phase eye position (SPEP) in responses to Slow and Fast HC. Group, patients vs. controls; direction, lesion vs. healthy side asymmetric rotation; time, testing time. Size effect: partial η^2^.

In conclusion, the main difference observed between the final position of the eye (VOR SPEP) and hand pointing (motion perception, FPE) was observed in the Slow HC responses toward the lesion side: over time, the reflex responses recovered almost completely whereas the perceptual responses recovered only partially (Fig. 3, *B* and *E*).

**Fig. 5.**
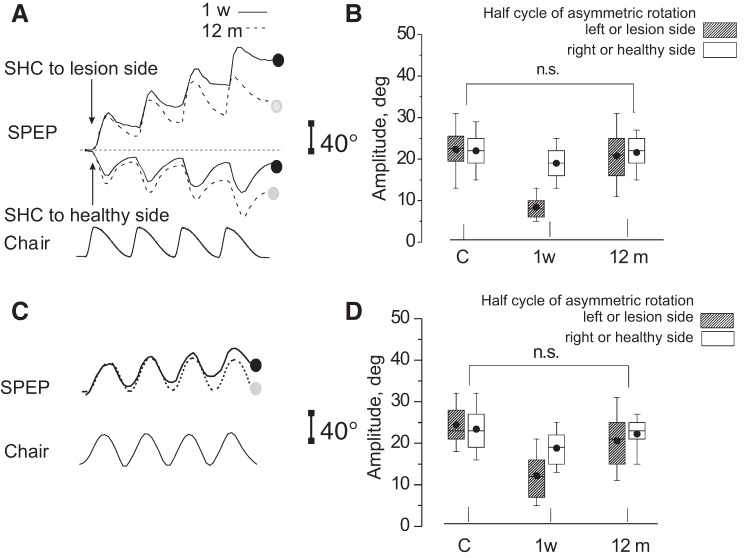
Continuous recording of eye position in 10 patients in the early and late phase of the compensation period. *A* and *C*: slow-phase eye position (SPEP) in response to opposite directed series of 4 asymmetric cycles (*A*) with Slow HC to lesion side (*top* traces, ↑) or to healthy side (*bottom* traces, ↓) and to symmetric rotation (*C*). In *A* and *C*: solid line represents 1 wk after the acute UVL episode and dashed line 12 mo after. Vertical bar: calibration of tracking. *Bottom* of *A* and *C* shows the chair rotation. Note the large difference in the cumulative SPEP [black spot: at 1 wk after the UVL acute episode (*A*) depending on the direction of the asymmetric rotation; at 12 mo there is no difference (gray spot)]. In response to symmetric rotation (*C*) SPEP, the directional difference was present only at 1 wk. *B* and *D*: graphs of the amplitude (in degrees) of the SPEP in response to the last Slow HC asymmetric (*B*) and symmetric rotation (*D*). Box, whisker, horizontal line and circle represent range, quartile, median, and mean, respectively. Dashed box, Slow HC rotation to left or lesion side; white box, Slow HC rotation to right or healthy side. The response obtained from the controls (C) and in UVL patients at 1 wk (1 w) and 12 mo (12 m). Note that there is no significant difference of the Slow HC of the asymmetric rotation at 12 mo between controls and UVL patients in response neither to asymmetric nor to symmetric rotation. Statistical data: asymmetric Slow HC to lesion side: groups (patients and controls) (*F*_1,18_ = 6.3, *P* < 0.01, = 0.33), time (*F*_1,18_ = 0.21, *P* = 0.35, partial η^2^ = 0.62), groups×time (*F*_1,18_ = 0.75, *P* = 0.78, partial η^2^ = 0.59); asymmetric Slow HC to healthy side: groups (*F*_1,18_ = 5.3, *P* < 0.05, partial η^2^ = 0.16), time (*F*_1,18_ = 0.73, *P* = 0.16, partial η^2^ = 0.77), groups×time (*F*_1,18_ = 1.9, *P* = 0.21, partial η^2^ = 0.31); symmetric Slow HC to lesion side: groups (patients and controls) (*F*_1,18_ = 5.2, *P* < 0.05, partial η^2^ = 0.48), time (*F*_1,18_ = 0.24, *P* = 0.38, partial η^2^ = 0.43), groups×time (*F*_1,18_ = 0.35, *P* = 0.46, partial η^2^ = 0.41); symmetric Slow HC to healthy side: groups (patients and controls) (*F*_1,18_ = 4.6, *P* < 0.05, partial η^2^ = 0.16), time (*F*_1,18_ = 0.45, *P* = 0.7, partial η^2^ = 0.30), groups×time (*F*_1,18_ = 0.58, *P* = 0.59, partial η^2^ = 0.31).

The mean amplitude of the responses to four asymmetric and symmetric (0.09 Hz) Slow HC rotations were also compared (Fig. 5 and statistical data in the figure legend). During asymmetric rotation the VOR SPEP was depressed in the early measures (*P* < 0.014). However, at 12 mo the responses fully recovered the control values (*P* = 0.3). The amplitude of the response to symmetric rotation was also reduced at the early measure and recovered to normal values in both directions at 12 mo (*P* = 0.3) (Fig. 5).

### Comparative Recovery of Motion Perception, Subjective Visual Vertical, Caloric, and Head-Shaking Responses

**Fig. 6.**
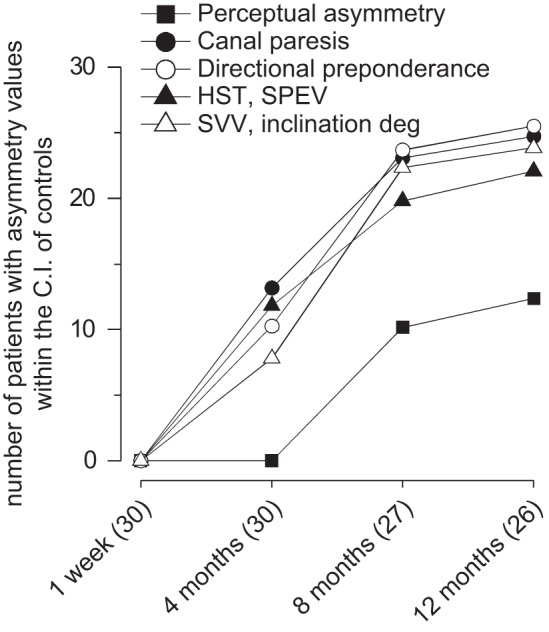
Recovery of vestibular symptoms to normal values. Number of patients showing values of the vestibular response asymmetry within the confidence interval (CI) of controls for perceptual asymmetry (■), canal paresis (●), directional preponderance (○), head shaking test (HST) slow-phase eye velocity (SPEV) (▲), and subjective visual vertical (SVV) inclination (△). In abscissa the testing time and the number of examined patients are shown within brackets.

**Fig. 7.**
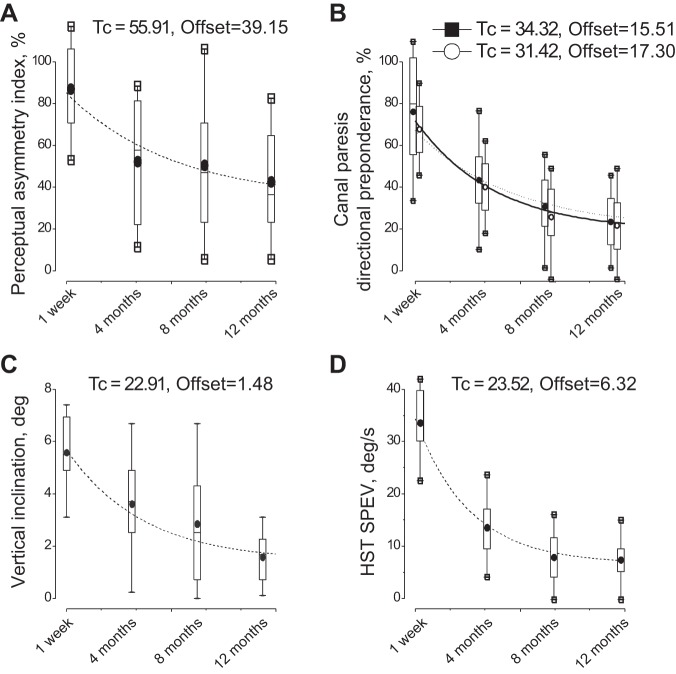
Time courses of the reflex and perceptual recovery after acute UVL in 30 patients. *A*: perceptual asymmetry index. *B*: caloric canal paresis (● and exponential decay, solid line) and caloric directional preponderance (○ and exponential decay, dotted line). *C*: inclination of subjective visual vertical. *D*: slow-phase eye velocity (SPEV) of the responses to head shaking test (HST). Box, whisker, horizontal line and circle represent quartile, range, median, and mean, respectively. Tc, time constant (express as days) and offset of the exponential decay (dotted and solid line). χ^2^ of the fitting: 25.21 (*A*), 21.19 (*B*), 51.67 (*C*), 39.59 (*D*).

We calculated the mean and 95% CIs for asymmetry in the control group in the perceptual (FPE), caloric, SVV, and head-shaking nystagmus tests. The asymmetry present in the patients’ results were then compared with those in control subjects. We found that the perceptual asymmetry in the patients was outside the CI of control group at any time after the acute episode. Even at 12 mo, 70% of patients showed values outside the control CI (Fig. 6). In contrast, for all other tests, the degree of asymmetry was outside the normal range in all patients only at 1 wk and 4 mo, but it returned within the normal range at 8 and 12 mo in 60–70% of patients. The GLM analysis for caloric, head shaking tests, and visual vertical tests confirms the difference in the recovery of perceptual and reflex responses: caloric test [groups (patients vs. controls): *F*_1,51_ = 46.5, *P* = 0.003, partial η^2^ = 0.76, testing time: *F*_3,153_ = 11.1, *P* = 0.04, partial η^2^ = 0.74, and interaction group×time: *F*_3,153_ = 16.9, *P* = 0.003, partial η^2^ = 0.78]; head shaking test [groups (patients vs. controls): *F*_1,51_ = 41.7, *P* = 0.006, partial η^2^ = 0.67), testing time *F*_3,153_ = 12.3, *P* = 0.03, partial η^2^ = 0.70, and interaction group×time: *F*_3,153_ = 15.4, *P* = 0.004, partial η^2^ = 0.64]; visual vertical [groups (patients vs. controls): *F*_1,51_ = 38.1, *P* = 0.008, partial η^2^ = 68), time *F*_3,153_ = 10.9, *P* = 0.02, partial η^2^ = 85, and interaction group×time: *F*_3,153_ = 13.3, *P* = 0.005, partial η^2^ = 0.76]. Post hoc analysis showed that the asymmetry values were similar to the control value at 8 or 12 mo (*P* = 0.7–0.2), whereas the asymmetry of perceptual responses persisted different from that of controls (*P* < 0.001). The different trend observed in symmetry recovery between the perceptual response and that in the conventional vestibular tests, was supported by the longer time constants of decay in the return-to-normal process of the former, as measured from exponential best fits (Fig. 7).

At 12 mo 46% of patients showed good recovery in conventional VOR testing (values within the CI), but persistent asymmetry in motion perception, 30% of patients good recovery of both VOR and perception, and 24% of patients poor recovery of VOR and persistent perceptual asymmetry.

It could be argued that the reason for the long-term asymmetry in motion perception is the presence of a persisting caloric abnormality in some of these patients. However, we plotted the perception results against those of conventional vestibular responses for each patient. We found a good correlation between the degree of motion perception asymmetry and that of the other responses at 1 wk. This linear correlation was very weak at 12 mo (Table 5) indicating that the reason for the persistence of the asymmetry in motion perception is not related to the presence of a reflex caloric abnormality.

**Table 5.** Correlation between self-motion perception asymmetry and values of canal paresis, directional preponderance, slow-phase eye velocity of head shaking test and inclination of subjective visual vertical in UVL patients at different testing time after acute episode

Measure time	Correlation	*R* Value	*P* Value
1 wk	SMP vs. CP	0.66	*P* < 0.012
	SMP vs. DP	0.71	*P* < 0.023
	SMP vs. HST	0.63	*P* < 0.031
	SMP vs. SVV	0.62	*P* < 0.036
4 mo	SMP vs. CP	0.31	*P* < 0.014
	SMP vs. DP	0.28	*P* < 0.021
	SMP vs. HST	0.33	*P* < 0.001
	SMP vs. SVV	0.15	*P* = 0.37
8 mo	SMP vs. CP	0.21	*P* < 0.02
	SMP vs. DP	0.13	*P* < 0.043
	SMP vs. HST	0.23	*P* < 0.021
	SMP vs. SVV	0.05	*P* = 0.13
12 mo	SMP vs. CP	0.18	*P* < 0.045
	SMP vs. DP	0.11	*P* < 0.039
	SMP vs. HST	0.05	*P* = 0.12
	SMP vs. SVV	0.01	*P* = 0.27

*R* and *P* of regression fitting curve between the variable self-motion perception (SMP), caloric test (CP, canal paresis; DP, directional preponderance), head shaking test (HST), and subjective visual vertical (SVV) at 1 wk and 4, 8, and 12 mo after acute episode of with unilateral vestibular lesions (UVL) in 30 patients.

### Correlation Between DHI Scores, Self-Motion Perception, and Conventional Vestibular Tests

**Fig. 8.**
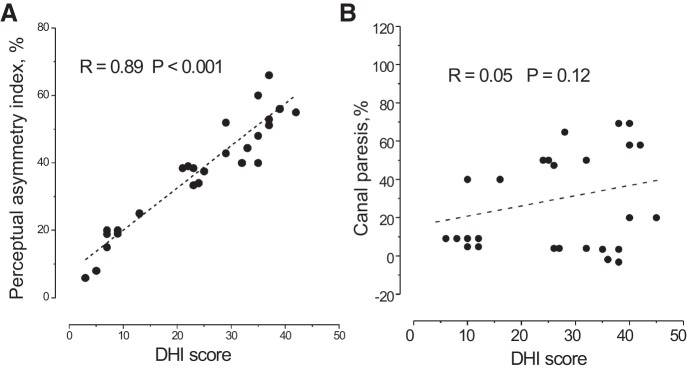
Correlation of perceptual and reflex asymmetries with DHI score at 12 mo. *A*: correlation between DHI score and motion perception asymmetry. *B*: correlation between DHI score and canal paresis. Correlation coefficient (*R*) and probability (*P*).

Patient discomfort or symptom load as measured with the DHI was evaluated at 12 mo after the acute episode. A moderate handicap persisted with a mean score of 28 points, compatible with published long-term DHI values in UVL patients (Cousins et al. 2014). For each patient the DHI score was compared with the values of asymmetry of self-motion perception, caloric responses, SVV tilt and the head shaking test (Fig. 8, *A* and *B*; Table 6). The DHI score showed a very high correlation with the asymmetry index of self-motion perception (*R* = 0.89, *P* = 0.0009) but no correlation with canal paresis (*R* = 0.05, *P* = 0.12), slow-phase eye velocity after head shaking (*R* = −0.04, *P* = 0.69), and SVV tilt (*R* = −0.01, *P* = 0.46). Concerning the DHI subitems, physical impairment did not correlate with self-motion asymmetry (*R* = 0.06, *P* = 0.041), while the correlation was observed for the emotional (*R* = 0.82, *P* = 0.0017) and functional subscales (*R* = 0.85, *P* = 0.0019). The DHI evaluated in the 10 patients, in which continuous perceptual tracking and VOR were examined, was also highly correlated with the self-motion perception asymmetry index (*R* = 0.91, *P* = 0.001). In view of recent evidence (Arshad et al. 2013) we examined whether patient handedness had any effect on our results. As there were only six left-handed patients, the only meaningful comparison we could implement was whether results in right-handed patients differed between those with right- and left-sided vestibular lesion. No differences between these two subgroups were found for final position tracking error (*F* = 1.1, *P* = 0.52), DHI (*F* = 0.92, *P* = 0.26), or the correlation between these two parameters (right lesion: *R* = 0.88; left lesion: *R* = 0.89).

**Table 6.** Correlation between DHI and values of self-motion perception asymmetry, canal paresis, directional preponderance, slow-phase eye velocity of head shaking test, and inclination of Subjective Visual Vertical in UVL patients at 12 mo after acute episode

Correlation	*R* Value	*P* Value
DHI vs. SMP	0.89	*P* < 0.0009
DHI vs. CP	0.05	*P* = 0.12
DHI vs. DP	0.09	*P* = 0.15
DHI vs. HST	−0.04	*P* = 0.69
DHI vs. SVV	−0.01	*P* = 0.46

*R* and *P* of the of regression fitting curve between the variables Dizziness Handicap Inventory (DHI) and self-motion perception (SMP), caloric test (CP, canal paresis; DP, directional preponderance), head shaking test (HST), and subjective visual vertical (SVV) at 12 mo after the acute episode of unilateral vestibular lesions (UVL) in 30 patients.

## DISCUSSION

The main finding of this paper is the pronounced and long-term deficit in self-motion perception revealed by asymmetric rotation in UVL patients. Reflex vestibulo-ocular function, assessed by symmetric or asymmetric rotation or by conventional clinical vestibular tests, regains normality more quickly and more fully than self-motion perception. Of potential clinical interest, the degree of perceptual asymmetry correlates with clinical outcome.

### Asymmetry of Self-Motion Perception in UVL Patients

In patients the large FPE found depends on the interaction between the lesion and the different ability of the vestibular system to convert velocity into displacement at high and low frequencies. FPE is maximal when the Slow HC is toward the lesion side, when the slow motion is hardly perceived. FPE is minimal when fast rotation is toward the lesion side, probably because perception of the fast rotation is already degraded by the lesion and, in this way, counterbalances the underestimation of low-frequency rotation toward the intact side. As expected, in normal subjects, the FPEs are the same in either direction.

In a previous paper in normal subjects (Pettorossi et al. 2013) we showed that asymmetric rotation induces a vestibular adaptive mechanism that depresses the responses to the slow rotation. Thus the remarkable FPE asymmetry observed in UVL patients likely results not only from the different intensity of vestibular activation per se, but also from this adaptive effect induced by asymmetric stimulation. Notably, this adaptive mechanism maintains the FPE asymmetry even when peripheral recovery and central compensation have taken place, as shown by the efficient compensation of the VOR observed in most of patients. Furthermore, detailed examination of a subgroup of 10 relatively homogeneous patients (similar initial and final values of canal paresis) showed that the main effect of UVL was a decrease in sensitivity during the Slow HC rotation when directed toward the lesion side, selectively for perceptual but not VOR responses. Fast responses were less affected and gradually recovered during the compensatory period. Although this might be the result of a general postlesion increase in the proportion of irregular afferent units, shown in UVL monkeys (Sadeghi et al. 2007), a different central adaptive mechanism is more likely responsible for the persistent depression in the response to the slow rotation. This is strongly supported by two findings: first, that the reduction in sensitivity to slow rotation did not occur for the VOR and, second, that the persistent depression was not observed during slow rotation during symmetric stimulation. In fact, under symmetric rotation the perceptual gain gradually improved during the process of central vestibular compensation.

For similar reasons, we discarded the hypothesis that the persistent perceptual asymmetry observed in patients is mediated by changes in vestibular thresholds brought about by the vestibular lesion. Indeed, threshold symmetry is restored within 10 wk (Cousins et al. 2013). On the whole, we suggest that the effect of asymmetric rotation on central processing is responsible for the perceptual persistent asymmetry and that the preceding fast rotation is the critical factor responsible for keeping the gain of the slow responses down. When contrasting high and low frequencies are delivered in combination or in sequence, as with the stimulus used here, the perception of higher over lower frequency rotations is favored (Massot et al. 2012; Panichi et al. 2011; Pettorossi et al. 2013; Tremblay et al. 2013). This centrally mediated phenomenon due to frequency contrast selectively affects perception of self-motion but not reflex function such as the VOR, and our present results demonstrate that the loss of unilateral peripheral vestibular input preserves this central adaptive property. Thus the compensatory process triggered by a UVL seems to prioritize recovery of this unique function (preferential high-frequency perceptual sensitivity), perhaps to the detriment of the lesioned side being less able to perceive low-frequency rotations.

Although our findings in UVL patients are specific for vestibular neuritis, a limitation of this study is that clinical recovery in vestibular neuritis is due to a dual process of peripheral recovery of the presumably viral lesion and central compensation (unlike experimental animals with vestibular neurectomy, in whom recovery can only be due to central compensation). However, vestibular neurectomies (for refractory vertigo) have been abandoned. Furthermore, we noted that in several patients with values of canal paresis reaching the range of normality (including the 10 patients studied in detail), the perceptual responses remained significantly asymmetric. Accepting that the degree of caloric canal paresis is a measure of peripheral recovery (Strupp et al. 2004), it seems that the perceptual deficit persists even when the peripheral input is largely restored.

### Asymmetry of VOR in UVL Patients

In UVL patients the VOR behaves differently to the perceptual responses. Asymmetric rotation affects the cumulative ocular position only in the earlier stages of the vestibular lesion. This directional asymmetry gradually diminishes over time so that, in the final months, a clear divergence between perceptual and reflex responses is present. As mentioned, this divergence is mostly caused by the different vestibular sensitivity to the Slow HC directed toward the lesion side, which recovers for the VOR but not for self-motion perception.

In this regard, it is important to recall that normal subjects exposed to asymmetric rotation exhibit an oppositely directed adaptation process for the perceptual and reflex responses (Pettorossi et al. 2013). The cumulative eye position error at the end of prolonged asymmetric rotations is much less than the perceptual FPE and, furthermore, whereas perceptual responses to the Slow HC gradually decrease (becoming more asymmetric), VOR symmetry gradually increases (Pettorossi et al. 2013). This oppositely directed adaptive mechanism present during asymmetric stimulation in control subjects may underlie the increased reflex-perceptual dissociation observed in UVL patients.

A different type of vestibular ocular-perceptual dissociation in UVL was previously reported, showing that the perception of rotational velocity was attenuated more than VOR responses in the acute vertiginous phase; a kind of cortical “anti-vertiginous” protective mechanism (Cousins et al. 2013, 2017). This is in clear contrast to our present result as we now report a persistent motion perception abnormality that, owing to the fact that it correlates with dizziness handicap (see *Vestibular Perceptual Deficits and Symptoms: Clinical Implications* below), cannot be considered a protective or compensatory phenomenon.

### The Possible Role of Prediction and Attention on the Perceptual Asymmetry in UVL Patients

The findings during asymmetric rotation likely reflect the influence of an underlying adaptive process potentially useful for enhancing awareness of high-frequency components of self-motion (Massot et al. 2012; Panichi et al. 2011; Pettorossi et al. 2013; Tremblay et al. 2013). However, other possibilities need to be considered. Predictive mechanisms may be involved, particularly as the tracking task used could be regarded as (nonocular) smooth pursuit, which is known to be enhanced by prediction, particularly at low frequencies (Barnes et al. 1987). It may be possible that a decline of the predictive mechanism during low-frequency response could be at the base of the large perceptual asymmetry, but experiments comparing pursuit vs. vestibularly guided movements showed that the latter do not display predictive characteristics (Yasui and Young 1984). Moreover, a reduction of predictive mechanisms is unlikely since prediction is enhanced rather than depressed in humans with vestibular lesions as part of the central process of vestibular compensation (Kasai and Zee 1978; Weber et al. 2008).

Another mechanism that could influence the perceptual sensitivity to the Slow HC was suggested by a previous finding that, in normal subjects, head-on-trunk deviations of the face in the direction of the Fast HC increase the final tracking error further (Panichi et al. 2011). This effect, which may be driven by attentional mechanism, would make subjects focus on fast frequencies or accelerations in the direction of motion and impending straight-ahead. Alternatively, however, this modulation could be the result of a direct bottom-up effect of neck proprioception, rather than attentional, which unfortunately was not investigated in our UVL patients.

### Putative Neuronal Structures at the Basis of the Vestibuloperceptual Findings

The distinct time courses for motion perception and reflex normalization discussed above calls for separate central compensatory mechanisms in the brain. Early stages of the vestibular processing network, e.g., the vestibular nuclei, could be at least partly responsible for some of the dissociated effects observed. Vestibular-only neurons in the vestibular nuclei encoding self-motion perception present different characteristics than position vestibular-pause neurons encoding ocular responses (Cullen 2012). Similarly, in vitro studies (Grassi et al. 1995; Pettorossi et al. 2011) have shown that long-term potentiation or depression can be elicited in different areas of the medial vestibular nuclei depending on the different intensity of the vestibular afferent activation, which, in turn, might mediate the divergence in perceptual and reflex vestibular responses.

In addition to low brain stem circuitry, a number of higher order central nervous system structures are involved in vestibuloperceptual processing and, therefore, likely to be involved in the ocular-perceptual dissociation reported here. These include the cerebellum, thought to be a station in vestibulo-cortical pathways (Bronstein et al. 2008; Cullen 2012; Shaikh 2004), the hippocampus (Sharp et al. 1995), the posterior parietal cortex and parietoinsular vestibular cortex (Brandt and Dieterich 1999; Lopez and Blanke 2011), the posterior parietal cortex (Seemungal et al. 2008), and the neighboring temporoparietal junction (Kaski et al. 2016), particularly of the right hemisphere, that may be involved in the velocity-to-position integration process for vestibular perception. Indeed, structural MRI research shows that some of these areas show changes associated with central compensatory processes in UVL patients (Dieterich 2007; Helmchen et al. 2009).

### Vestibular Perceptual Deficits and Symptoms: Clinical Implications

The vestibular perceptual bias in UVL revealed by asymmetric rotation diminished over time although less so, and with a slower time course, than that found in reflex vestibular responses. After 12 mo the asymmetry of self-motion perception was still higher than the highest value observed in control subjects, whereas VOR-based functions such caloric reactivity, SVV tilt, and head shaking nystagmus results had all fallen within the normal range. The fact that caloric responses and the head shaking test (involving low- and high-frequency vestibular stimuli, respectively) recovered faster than the perceptual responses suggests that the whole spectrum of reflex responses behaved differently from perceptual responses.

Of note, the vestibular perceptual deficit elicited by asymmetric rotation seems relevant to the patients discomfort and handicap. This was shown by the significant correlation between the final tracking position error and the patients’ subjective vestibular handicap (DHI), a correlation not found with any other vestibular result. A unilateral vestibular deficit likely causes asymmetric signal activation in central vestibular structures and a mismatch between real and perceived motion, a mismatch that contributes to patients’ protracted symptoms of dizziness and spatial disorientation (Jáuregui-Renaud et al. 2008).

It could be argued that the deficit in slow motion perception reported here should not cause subjective discomfort to patients because sensory systems with better frequency response to low-frequency stimuli (vision, typically) could take over and make up for the deficit. However, it should be noted that the lesion-induced ocular-perceptual dissociation we report will inevitably create a mismatch between visual and vestibular afferent signals. In turn, it is well established that visuovestibular mismatch causes motion sickness even in control subjects (Cheung et al. 1991; Dai et al. 2011) and, therefore, such a mismatch between visual and vestibular head motion signals could easily contribute to the patients’ subjective symptoms. Psychological (Godemann et al. 2005) and visual perceptual factors (visual dependence; Cousins et al. 2014, 2017; Guerraz et al. 2001a, 2001b, 2001c) are also involved in the persistent discomfort of some UVL patients. However, in contrast to previous reports (Cousins et al. 2017) we have now identified a purely vestibular self-motion perceptual deficit being associated with patients’ long-term clinical outcome.

### Conclusions

Asymmetric rotation (Pettorossi et al. 2013) allowed us to establish that self-motion perception is profoundly disrupted by UVL and that its central compensation is slower and less complete than those of other aspects of vestibular function. The deficit is specific for the perceptual task during asymmetric rotation and the mechanisms responsible appear to be related to central adaptive processes favoring perception of fast over slow rotations. Different pathways involved in central vestibular compensation for vestibulo-ocular vs. vestibuloperceptual functions likely underlie these findings. Self-motion perception does not fully recover after 1 yr from the acute UVL episode and such delayed motion perception recovery may be partly responsible for patients’ discomfort.

## GRANTS

This research was supported by grants of Fondazione CRP 2015 and Ministero della Salute RF2011-02352379. A. M. Bronstein received support from the UK Medical Research Council and Biomedical Research Centre National Institute of Health Research.

## DISCLOSURES

No conflicts of interest, financial or otherwise, are declared by the authors.

## AUTHOR CONTRIBUTIONS

M.F., A.M.B., and V.E.P. conceived and designed research; R.P., A.K., C.O., and A.F. performed experiments; R.P., M.F., A.K., C.O., A.F., and V.E.P. analyzed data; R.P., M.F., R.B., A.K., C.O., A.F., A.M.B., and V.E.P. approved final version of manuscript; M.F., R.B., A.K., C.O., A.F., A.M.B., and V.E.P. interpreted results of experiments; M.F., A.M.B., and V.E.P. drafted manuscript; M.F., R.B., C.O., A.M.B., and V.E.P. edited and revised manuscript; A.F. prepared figures.

## References

[B1] Adamec I, Krbot Skorić M, Ozretić D, Habek M Predictors of development of chronic vestibular insufficiency after vestibular neuritis. J Neurol Sci 347: 224–228, 2014. doi:10.1016/j.jns.2014.10.001. 25307984

[B2] Ahmad H, Cerchiai N, Mancuso M, Casani AP, Bronstein AM Are white matter abnormalities associated with “unexplained dizziness”? J Neurol Sci 358: 428–431, 2015. doi:10.1016/j.jns.2015.09.006. 26412160 PMC4640145

[B3] Angelaki DE, Cullen KE Vestibular system: the many facets of a multimodal sense. Annu Rev Neurosci 31: 125–150, 2008. doi:10.1146/annurev.neuro.31.060407.125555. 18338968

[B4] Arshad Q, Nigmatullina Y, Bronstein AM Handedness-related cortical modulation of the vestibular-ocular reflex. J Neurosci 33: 3221–3227, 2013. doi:10.1523/JNEUROSCI.2054-12.2013. 23407975 PMC6619206

[B5] Baloh RW Vestibular neuritis. N Engl J Med 348: 1027–1032, 2003. doi:10.1056/NEJMcp021154. 12637613

[B6] Barnes GR, Donnelly SF, Eason RD Predictive velocity estimation in the pursuit reflex response to pseudo-random and step displacement stimuli in man. J Physiol 389: 111–136, 1987. doi:10.1113/jphysiol.1987.sp016649. 3681722 PMC1192073

[B7] Bergenius J, Perols O Vestibular neuritis: a follow-up study. Acta Otolaryngol 119: 895–899, 1999. doi:10.1080/00016489950180243. 10728930

[B8] Bloomberg J, Melvill Jones G, Segal B Adaptive plasticity in the gaze stabilizing synergy of slow and saccadic eye movements. Exp Brain Res 84: 35–46, 1991. doi:10.1007/BF00231760. 1855563

[B10] Brandt T, Dieterich M The vestibular cortex. Its locations, functions, and disorders. Ann N Y Acad Sci 871: 293–312, 1999. doi:10.1111/j.1749-6632.1999.tb09193.x. 10372080

[B11] Bronstein AM, Grunfeld EA, Faldon M, Okada T Reduced self-motion perception in patients with midline cerebellar lesions. Neuroreport 19: 691–693, 2008. doi:10.1097/WNR.0b013e3282fbf9f6. 18382289

[B12] Cheung BS, Howard IP, Money KE Visually-induced sickness in normal and bilaterally labyrinthine-defective subjects. Aviat Space Environ Med 62: 527–531, 1991. 1859339

[B13] Cousins S, Cutfield NJ, Kaski D, Palla A, Seemungal BM, Golding JF, Staab JP, Bronstein AM Visual dependency and dizziness after vestibular neuritis. PLoS One 9: e105426, 2014. doi:10.1371/journal.pone.0105426. 25233234 PMC4169430

[B68] Cousins S, Kaski D, Cutfield N, Arshad Q, Ahmad H, Gresty MA, Seemungal B, Golding J, Bronstein AM Predictors of clinical recovery from vestibular neuritis: a prospective study. Ann Clin Transl Neurol 4: 340–346, 2017. doi:10.1002/acn3.386. 28491901 PMC5420806

[B14] Cousins S, Kaski D, Cutfield N, Seemungal B, Golding JF, Gresty M, Glasauer S, Bronstein AM Vestibular perception following acute unilateral vestibular lesions. PLoS One 8: e61862, 2013. doi:10.1371/journal.pone.061862. 23671577 PMC3650015

[B15] Cullen KE The vestibular system: multimodal integration and encoding of self-motion for motor control. Trends Neurosci 35: 185–196, 2012. doi:10.1016/j.tins.2011.12.001. 22245372 PMC4000483

[B16] Curthoys IS Vestibular compensation and substitution. Curr Opin Neurol 13: 27–30, 2000. doi:10.1097/00019052-200002000-00006. 10719646

[B17] Dai M, Raphan T, Cohen B Prolonged reduction of motion sickness sensitivity by visual-vestibular interaction. Exp Brain Res 210: 503–513, 2011. doi:10.1007/s00221-011-2548-8. 21287155 PMC3182575

[B18] Dieterich M Functional brain imaging: a window into the visuo-vestibular systems. Curr Opin Neurol 20: 12–18, 2007. doi:10.1097/WCO.0b013e328013f854. 17215683

[B20] Dutia MB Mechanisms of vestibular compensation: recent advances. Curr Opin Otolaryngol Head Neck Surg 18: 420–424, 2010. doi:10.1097/MOO.0b013e32833de71f. 20693901

[B21] Faralli M, Ricci G, Molini E, Longari F, Altissimi G, Frenguelli A Determining subjective visual vertical: dynamic versus static testing. Otol Neurotol 28: 1069–1071, 2007. doi:10.1097/MAO.0b013e31815aea1b. 18084818

[B22] Friedmann G The judgement of the visual vertical and horizontal with peripheral and central vestibular lesions. Brain 93: 313–328, 1970. doi:10.1093/brain/93.2.313. 5310320

[B23] Godemann F, Koffroth C, Neu P, Heuser I Why does vertigo become chronic after neuropathia vestibularis? Psychosom Med 66: 783–787, 2004. doi:10.1097/01.psy.0000140004.06247.c9. 15385707

[B24] Godemann F, Siefert K, Hantschke-Brüggemann M, Neu P, Seidl R, Ströhle A What accounts for vertigo one year after neuritis vestibularis — anxiety or a dysfunctional vestibular organ? J Psychiatr Res 39: 529–534, 2005. doi:10.1016/j.jpsychires.2004.12.006. 15992562

[B25] Grabherr L, Nicoucar K, Mast FW, Merfeld DM Vestibular thresholds for yaw rotation about an earth-vertical axis as a function of frequency. Exp Brain Res 186: 677–681, 2008. doi:10.1007/s00221-008-1350-8. 18350283

[B26] Grassi S, Della Torre G, Capocchi G, Zampolini M, Pettorossi VE The role of GABA in NMDA-dependent long term depression (LTD) of rat medial vestibular nuclei. Brain Res 699: 183–191, 1995. doi:10.1016/0006-8993(95)00895-W. 8616620

[B27] Guerraz M, Gianna CC, Burchill PM, Gresty MA, Bronstein AM Effect of visual surrounding motion on body sway in a three-dimensional environment. Percept Psychophys 63: 47–58, 2001a. doi:10.3758/BF03200502. 11304016

[B28] Guerraz M, Thilo KV, Bronstein AM, Gresty MA Influence of action and expectation on visual control of posture. Brain Res Cogn Brain Res 11: 259–266, 2001b. doi:10.1016/S0926-6410(00)00080-X. 11275487

[B29] Guerraz M, Yardley L, Bertholon P, Pollak L, Rudge P, Gresty MA, Bronstein AM Visual vertigo: symptom assessment, spatial orientation and postural control. Brain 124: 1646–1656, 2001c. doi:10.1093/brain/124.8.1646. 11459755

[B30] Halmagyi GM, Weber KP, Curthoys IS Vestibular function after acute vestibular neuritis. Restor Neurol Neurosci 28: 37–46, 2010. doi:10.3233/RNN-2010-0533. 20086281

[B31] Helmchen C, Klinkenstein J, Machner B, Rambold H, Mohr C, Sander T Structural changes in the human brain following vestibular neuritis indicate central vestibular compensation. Ann N Y Acad Sci 1164: 104–115, 2009. doi:10.1111/j.1749-6632.2008.03745.x. 19645887

[B32] Jacobson GP, Calder JA, Rupp KA, Shepherd VA, Newman CW Reappraisal of the monothermal warm caloric screening test. Ann Otol Rhinol Laryngol 104: 942–945, 1995. doi:10.1177/000348949510401205. 7492065

[B33] Jacobson GP, Newman CW The development of the Dizziness Handicap Inventory. Arch Otolaryngol Head Neck Surg 116: 424–427, 1990. doi:10.1001/archotol.1990.01870040046011. 2317323

[B34] Jáuregui-Renaud K, Sang FY, Gresty MA, Green DA, Bronstein AM Depersonalisation/derealisation symptoms and updating orientation in patients with vestibular disease. J Neurol Neurosurg Psychiatry 79: 276–283, 2008. doi:10.1136/jnnp.2007.122119. 17578858

[B69] Jongkees LBW Thermic test and electronystagmography. Acta Otorhinolaryngol Belg 19: 455–464, 1965. 5830227

[B36] Kamei T, Kimura K, Kaneko H, Noro H Revaluation of the head shaking test as a method of nystagmus provocation. Nippon Jibiinkoka Gakkai Kaiho 67: 1530–1534, 1964. doi:10.3950/jibiinkoka.67.11_1530. 14279164

[B37] Kasai T, Zee DS Eye-head coordination in labyrinthine-defective human beings. Brain Res 144: 123–141, 1978. doi:10.1016/0006-8993(78)90439-0. 638756

[B38] Kaski D, Quadir S, Nigmatullina Y, Malhotra PA, Bronstein AM, Seemungal BM Temporoparietal encoding of space and time during vestibular-guided orientation. Brain 139: 392–403, 2016. doi:10.1093/brain/awv370. 26719385 PMC4805090

[B39] Kim HA, Hong JH, Lee H, Yi HA, Lee SR, Lee SY, Jang BC, Ahn BH, Baloh RW Otolith dysfunction in vestibular neuritis: recovery pattern and a predictor of symptom recovery. Neurology 70: 449–453, 2008. doi:10.1212/01.wnl.0000297554.21221.a0. 18250289

[B40] Lopez C, Blanke O The thalamocortical vestibular system in animals and humans. Brain Res Brain Res Rev 67: 119–146, 2011. doi:10.1016/j.brainresrev.2010.12.002. 21223979

[B41] Massot C, Schneider AD, Chacron MJ, Cullen KE The vestibular system implements a linear-nonlinear transformation in order to encode self-motion. PLoS Biol 10: e1001365, 2012. doi:10.1371/journal.pbio.1001365. 22911113 PMC3404115

[B42] Nakamura T, Bronstein AM The perception of head and neck angular displacement in normal and labyrinthine-defective subjects. A quantitative study using a ‘remembered saccade’ technique. Brain 18: 1157–1168, 1995. 7496777 10.1093/brain/118.5.1157

[B43] Nola G, Mostardini C, Salvi C, Ercolani AP, Ralli G Validity of Italian adaptation of the Dizziness Handicap Inventory (DHI) and evaluation of the quality of life in patients with acute dizziness. Acta Otorhinolaryngol Ital 30: 190, 2010. 21253284 PMC3008147

[B44] Palla A, Straumann D, Bronstein AM Vestibular neuritis: vertigo and the high-acceleration vestibulo-ocular reflex. J Neurol 255: 1479–1482, 2008. doi:10.1007/s00415-008-0935-2. 18604466

[B45] Panichi R, Botti FM, Ferraresi A, Faralli M, Kyriakareli A, Schieppati M, Pettorossi VE Self-motion perception and vestibulo-ocular reflex during whole body yaw rotation in standing subjects: the role of head position and neck proprioception. Hum Mov Sci 30: 314–332, 2011. doi:10.1016/j.humov.2010.10.005. 21277644

[B46] Patel M, Arshad Q, Roberts RE, Ahmad H, Bronstein AM Chronic symptoms after vestibular neuritis and the high-velocity vestibulo-ocular reflex. Otol Neurotol 37: 179–184, 2016. doi:10.1097/MAO.0000000000000949. 26719963 PMC4712355

[B47] Pettorossi VE, Dieni CV, Scarduzio M, Grassi S Long-term potentiation of synaptic response and intrinsic excitability in neurons of the rat medial vestibular nuclei. Neuroscience 187: 1–14, 2011. doi:10.1016/j.neuroscience.2011.04.040. 21539898

[B48] Pettorossi VE, Panichi R, Bambagioni D, Grassi S, Botti FM Contribution of eye position to movement perception. Acta Otolaryngol 124: 471–474, 2004. doi:10.1080/00016480410017314. 15224877

[B49] Pettorossi VE, Panichi R, Botti FM, Biscarini A, Filippi GM, Schieppati M Long-lasting effects of neck muscle vibration and contraction on self-motion perception of vestibular origin. Clin Neurophysiol 126: 1886–1900, 2015. doi:10.1016/j.clinph.2015.02.057. 25812729

[B50] Pettorossi VE, Panichi R, Botti FM, Kyriakareli A, Ferraresi A, Faralli M, Schieppati M, Bronstein AM Prolonged asymmetric vestibular stimulation induces opposite, long-term effects on self-motion perception and ocular responses. J Physiol 591: 1907–1920, 2013. doi:10.1113/jphysiol.2012.241182. 23318876 PMC3624859

[B51] Pettorossi VE, Schieppati M Neck proprioception shapes body orientation and perception of motion. Front Hum Neurosci 8: 895, 2014. doi:10.3389/fnhum.2014.00895. 25414660 PMC4220123

[B52] Popov KE, Lekhel H, Faldon M, Bronstein AM, Gresty MA Visual and oculomotor responses induced by neck vibration in normal subjects and labyrinthine-defective patients. Exp Brain Res 128: 343–352, 1999. doi:10.1007/s002210050854. 10501806

[B53] Sadeghi SG, Minor LB, Cullen KE Response of vestibular-nerve afferents to active and passive rotations under normal conditions and after unilateral labyrinthectomy. J Neurophysiol 97: 1503–1514, 2007. doi:10.1152/jn.00829.2006. 17122313

[B55] Seemungal BM, Gunaratne IA, Fleming IO, Gresty MA, Bronstein AM Perceptual and nystagmic thresholds of vestibular function in yaw. J Vestib Res 14: 461–466, 2004. 15735328

[B56] Seemungal BM, Rizzo V, Gresty MA, Rothwell JC, Bronstein AM Posterior parietal rTMS disrupts human Path Integration during a vestibular navigation task. Neurosci Lett 437: 88–92, 2008. doi:10.1016/j.neulet.2008.03.067. 18440143

[B57] Shaikh AG, Meng H, Angelaki DE Multiple reference frames for motion in the primate cerebellum. J Neurosci 24: 4491–4497, 2004. doi:10.1523/JNEUROSCI.0109-04.2004. 15140919 PMC6729386

[B58] Sharp PE, Blair HT, Etkin D, Tzanetos DB Influences of vestibular and visual motion information on the spatial firing patterns of hippocampal place cells. J Neurosci 15: 173–189, 1995. 7823128 10.1523/JNEUROSCI.15-01-00173.1995 PMC6578264

[B59] Shupak A, Issa A, Golz A, Kaminer M, Braverman I Prednisone treatment for vestibular neuritis. Otol Neurotol 29: 368–374, 2008. doi:10.1097/MAO.0b013e3181692804. 18317392

[B60] Siegle JH, Campos JL, Mohler BJ, Loomis JM, Bülthoff HH Measurement of instantaneous perceived self-motion using continuous pointing. Exp Brain Res 195: 429–444, 2009. doi:10.1007/s00221-009-1805-6. 19396591

[B61] Smith PF, Curthoys IS Mechanisms of recovery following unilateral labyrinthectomy: a review. Brain Res Brain Res Rev 14: 155–180, 1989. doi:10.1016/0165-0173(89)90013-1. 2665890

[B62] Strupp M, Brandt T Vestibular neuritis. Semin Neurol 29: 509–519, 2009. doi:10.1055/s-0029-1241040. 19834862

[B63] Strupp M, Zingler VC, Arbusow V, Niklas D, Maag KP, Dieterich M, Bense S, Theil D, Jahn K, Brandt T Methylprednisolone, valacyclovir, or the combination for vestibular neuritis. N Engl J Med 351: 354–361, 2004. doi:10.1056/NEJMoa033280. 15269315

[B64] Tremblay L, Kennedy A, Paleressompoulle D, Borel L, Mouchnino L, Blouin J Biases in the perception of self-motion during whole-body acceleration and deceleration. Front Integr Neurosci 7: 90, 2013. doi:10.3389/fnint.2013.00090. 24379764 PMC3864246

[B65] Valko Y, Lewis RF, Priesol AJ, Merfeld DM Vestibular labyrinth contributions to human whole-body motion discrimination. J Neurosci 32: 13537–13542, 2012. doi:10.1523/JNEUROSCI.2157-12.2012. 23015443 PMC3467969

[B66] Weber KP, Aw ST, Todd MJ, McGarvie LA, Curthoys IS, Halmagyi GM Head impulse test in unilateral vestibular loss: vestibulo-ocular reflex and catch-up saccades. Neurology 70: 454–463, 2008. doi:10.1212/01.wnl.0000299117.48935.2e. 18250290

[B67] Yasui S, Young LR On the predictive control of foveal eye tracking and slow phases of optokinetic and vestibular nystagmus. J Physiol 347: 17–33, 1984. doi:10.1113/jphysiol.1984.sp015050. 6707954 PMC1199431

